# An optimized method for cryogenic storage of *Xenopus* sperm to maximise the effectiveness of research using genetically altered frogs

**DOI:** 10.1016/j.theriogenology.2017.01.007

**Published:** 2017-04-01

**Authors:** Esther Pearl, Sean Morrow, Anna Noble, Adelaide Lerebours, Marko Horb, Matthew Guille

**Affiliations:** a National Xenopus Resource, 7 MBL Street, Woods Hole, MA, 02543, USA; b European Xenopus Resource Centre, Institute of Biomedical and Biomolecular Sciences, University of Portsmouth, Portsmouth, PO1 2DT, UK; c School of Biological Sciences, University of Portsmouth, Portsmouth, PO1 2DY, UK

**Keywords:** *Xenopus*, Sperm, Cryopreservation, Stock centres, Genetically altered lines, 3Rs

## Abstract

Cryogenic storage of sperm from genetically altered *Xenopus* improves cost effectiveness and animal welfare associated with their use in research; currently it is routine for *X. tropicalis* but not reliable for *X. laevis*. Here we compare directly the three published protocols for *Xenopus* sperm freeze-thaw and determine whether sperm storage temperature, method of testes maceration and delays in the freezing protocols affect successful fertilisation and embryo development in *X. laevis*. We conclude that the protocol is robust and that the variability observed in fertilisation rates is due to differences between individuals. We show that the embryos made from the frozen-thawed sperm are normal and that the adults they develop into are reproductively indistinguishable from others in the colony. This opens the way for using cryopreserved sperm to distribute dominant genetically altered (GA) lines, potentially saving travel-induced stress to the male frogs, reducing their numbers used and making *Xenopus* experiments more cost effective.

## Highlights

• Xenopus cryopreservation is robust using an optimized method.
• Success is dependent on the quality of animals from which the sperm are taken.
• Frozen sperm may now be used to distribute lines and wild-type male gametes around the world.

## 1 Introduction

Cryogenic storage of germ cells or embryos from genetically altered, vertebrate model organisms has a key role in making the use of these models cost effective and efficient. It also improves animal welfare by reducing the number of animals held in stock centres and laboratories [1], [2], [3]. For amphibia, which are under great threat in the wild [4], [5], [6], [7], cryogenic storage may also have a role in preserving genetic diversity of threatened species [8].

Clawed frogs of the species *Xenopus laevis* and *Xenopus tropicalis* are arguably the most versatile vertebrate model organisms; they are used widely for basic cell and developmental biology [9] and more recently for the study of human genetic disorders [10], [11]. Alongside the traditional methods used in *Xenopus*, for example: explants [12], [13], [14], [15], morphant knockdowns [16], [17], [18], [19], [20], extract biochemistry [21], [22], [23], [24] and gain or loss of function screens [25], genetically altered lines have been made for 20 years [26], [27], [28], [29]. These include not only transgenic lines but also those produced from forward mutation screens [30], [31] and most recently by genome editing [32], [33], [34], [35], [36], [37], [38]. Although most of the use of *Xenopus* as a traditional genetic model organism has been confined to the diploid and short generation time frog, *Xenopus tropicalis*, gene editing is so efficient that it can be successfully applied to the allotetraploid *Xenopus laevis* [37], [38], [39], [40], which for reasons of robustness and history is more widely used.

Both model *Xenopus* species have the advantage of very long reproductively active lives, extending well over a decade [41] so it is not essential to keep lines frozen to avoid generational genetic drift [42]. Nonetheless keeping large numbers of *Xenopus* lines is costly due to the space and husbandry requirements and undesirable due to the animal welfare cost of keeping numbers of frogs in captivity over very long periods. At the European and US stock centres we hold more than 200 transgenic lines and even more mutant ones, they are duplicated at each centre to ensure their security [43]; these numbers are now increasing significantly as the first gene edited lines start to arrive. For these reasons the resource centres have been storing sperm cryogenically and, whilst this has been successful for *X. tropicalis*, the results with *X. laevis* have been much more inconsistent and we currently do not risk storing the *X. laevis* lines cryogenically. Recent studies on spermatozoon damage during cryopreservation [44] have shown that *X. laevis* samples undergo much more plasma membrane damage than those from *X. tropicalis* and this is a likely reason for the difference.

There are three major methods for cryopreservation of *Xenopus* spermatozoa: Sargent and Mohun [45], Mansour et al. [46] and that from the Harland laboratory website [47]. The method of Mansour and co-workers used motility inhibiting saline as a cryoprotectant and freezing in liquid nitrogen vapour; Sargent and Mohun used a sucrose and egg yolk-based cryoprotectant and freezing on a dry ice ethanol bath and the Harland lab method again used a sucrose and egg yolk-based cryoprotectant but with slow freezing in a styrofoam box placed into a −80C freezer. The aim of this study was to compare these methods using the same sperm and eggs, since the quality of these can influence results enormously, and then to test the effect of a number of parameters on the fertilisation capability of frozen-thawed sperm. Finally, we use these data to develop a robust method that can be adopted by the entire *Xenopus* research community.

## 2 Materials and methods

### 2.1 Maintaining frogs

All work involving animals was approved by the local AWERB or IACUC as appropriate, and in the UK it was carried out under the relevant Home Office legislation. Adult, outbred *X. laevis* were maintained at 18C in recirculating water with a 10% daily change. They were fed at least twice daily on Horizon XP high protein trout pellets. Eggs were produced as described in Ref. [48]. Briefly this entails a 500–800U injection of Human Chorionic Gonadotrophin (HCG) into a sexually mature female frog the night before the eggs are required. The following day the female frog is given a gentle abdomen massage to encourage her to lay eggs. Adult, outbred Nigerian *X. tropicalis* were kept similarly but at 24.5C.

The sperm cryopreservation experiments were performed on *X. tropicalis* using the Harland method, and on *X. laevis* using Sargent and Mohun, Mansour and Harland methods (see below for details of each method).

### 2.2 Masceration and homogenisation of testis

Fresh testes from the same males that were cryopreserved were stored for use as controls at 4C in 1 × Modified Barth's Saline (MBS: 88 mM NaCl, 0.41 mM CaCl_2_, 0.82 mM MgSO_4_, 10 mM HEPES, 0.41 mM Ca(NO_3_)_2_, 2.4 mM NaHCO_3)_; we found no evidence that such storage for up to a week, which is standard in most *Xenopus* labs, altered the outcomes of fertilisation. Two different methods of dissociating testis were used. Homogenising was performed by squashing the testicles in a microcentrifuge tube with a plastic pestle (EXRC method). Mascerating testicles was performed by tearing the testicles apart with two pairs of forceps (NXR method). The effect of method of testicle dissociation on fertilisation rate was tested. After dissociation of the testicles the sample is not purified, and thus it contains other cell types as well as sperm, but the dissociated sample is referred to as sperm. In order to quickly move from dissociated testis to the freezing process the concentration of the sperm in each sample was not quantified.

### 2.3 Sargent and Mohun sperm cryopreservation method

This method was published in 2005 [45]. Each testis was mascerated by tearing apart with forceps in ice-cold 500 μL L15 with glutaMAX, the ice cold cryoprotectant solution was added, and the resulting solution was divided evenly between 4 cryotubes. Unlike in Sargent and Mohun [45], samples were frozen in cryotubes to enable storage in liquid nitrogen (USA scientific catalog number 1405–9302). The freezing procedure used was the improvised apparatus mentioned by Sargent and Mohun [45]. This consisted of a plastic box with a tube rack filled with 300 mL ethanol and a magnetic stir bar, placed inside a larger box containing dry ice and ethanol, which had been set up on a stir plate and allowed to reach constant temperature. The temperature decreased 30 °C in 3 min. After 10 min in the tube rack in the central box samples were transferred to either the −80C freezer or a liquid nitrogen dewar for storage. Thawing was performed differently to the published method (see sperm thawing method section below), which was thawing at 30C for <10s.

### 2.4 Mansour sperm cryopreservation method

This method was published in 2009 [46]. The cryoprotectant used was motility inhibiting saline (MIS), the most effective of those tested by Mansour et al. [46], consisting of 150 mM NaCl, 3 mM KCl, 1 mM Mg_2_SO_4_, 1 mM CaCl_2_, and 20 mM Tris pH 8 with 5% DMSO and 73 mM sucrose. Each testis was macerated by tearing apart with forceps in 500 μL of ice-cold MIS, and the resulting macerate was divided evenly between 4 cryotubes. Samples were frozen in cryotubes for 10 min suspended 10 cm above the surface of liquid nitrogen, using an improvised rack devised from bent paperclips. The temperature decreased 60C in 3 min. Samples were then transferred to either the −80C freezer or a liquid nitrogen Dewar for storage. Thawing was performed differently to the published method (see sperm thawing method section below), which was at room temperature for 40s.

### 2.5 Harland sperm cryopreservation method

The Harland method uses a cryoprotectant similar to that of the Sargent method, the main difference being that it has half the concentration of NaHCO_3_ and sucrose in the cryoprotectant. Each testis was mascerated by tearing apart with forceps in ice-cold 500 μL L15 with glutamine, the ice cold cryoprotectant solution was added, and the resulting solution was divided evenly between 4 cryotubes. The samples were frozen by placing aliquots in tube racks in a room temperature Styrofoam box, which was then placed in the −80C freezer. After 24 h at −80C the sperm was either stored in racks at −80C or transferred to a liquid nitrogen Dewar. Thawing was performed differently to the published method (see sperm thawing method section below).

### 2.6 Sperm thawing method

Eggs were extracted from female frogs by gentle massage before thawing the sperm. Eggs were extracted before thawing sperm since activated motile sperm have a ‘motility half-life’ of 2 min [49]. In practice, thawing sperm stored in either −80C or liquid nitrogen took longer than any published protocols suggested. All sperm thawing, regardless of method of freezing, involved 40 s in a 37C water bath. Two to three volumes of 0.1 × Marc's Modified Ringers: 0.1 M NaCl, 2 mM KCl, 1 mM MgSO_4_, 2 mM CaCl_2_, 5 mM HEPES pH7.5, 0.1 mM EDTA (MMR) was added to the sperm as soon as about half of the frozen sperm had thawed. This was then pipetted up and down gently using a wide bore disposable transfer pipet. Sperm was then applied to eggs immediately all ice was gone from the solution; a minimum of 50 eggs were used for each sample.

### 2.7 Embryo culture

Fertilisation rates were assayed at NF3 (4-cell), embryos that had divided were counted as fertilised. Embryos were cultured at 18C in 90 mm petri dishes in 0.1 × MMR containing gentamicin (50μg/ml) at a density of 100 embryos or less per dish. Embryos were moved into clean petri dishes with fresh 0.1 × MMR every day. The number of live embryos were counted daily, as were the number of abnormal embryos. Embryos that deviated from the images shown in the normal table of *Xenopus* development were scored as abnormal. Photographs of representative normal and abnormal embryos were taken at NF32 (Nieuwkoop and Faber stage 32) [50].

### 2.8 Whole mount in situ hybridisation

All probes were obtained from the EXRC's collection and the method followed was that of Broadbent and Read [51].

### 2.9 Statistical analyses

All experiments used a minimum of 3 replicates for sperm and 2 for eggs, more than 50 embryos were used as biological replicates. Statistical analyses were performed using R 3.0 [52]. The effects of the species, method (Mansour, Harland and Sargent), time, technique (homogenisation or masceration), delay post-thawing, maceration delay, testes division and the cryostorage on the % fertility, % normal development and % survival to NF32 were tested using linear models (anova.lm). The normality of the residuals was checked using the Shapiro-Wilk normality test. The data were Arcsin transformed when necessary. The differences between the 3 cryopreservation methods on the % of fertilisation were assessed using the contrast method [53] with the Esticon function in R (DoBy package) [54].

## 3 Results

### 3.1 Sperm cryopreservation is successful in X. tropicalis but inconsistent in *X. laevis*

**Fig. 1:**
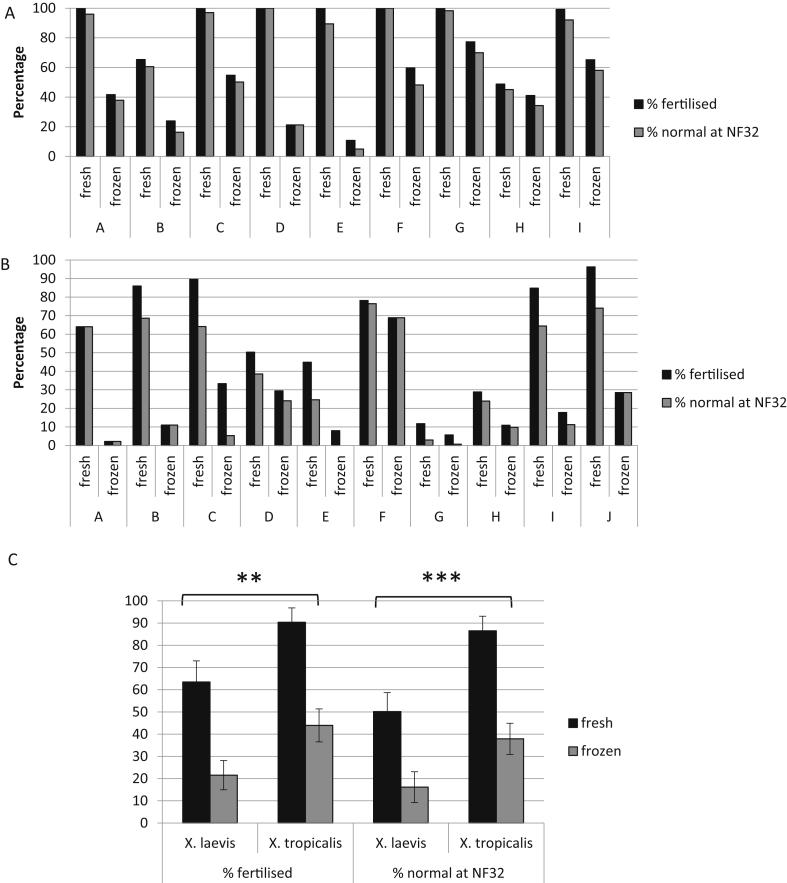
**Cryogenic preservation of *X. laevis* sperm is less effective and consistent than for *X. tropicalis* sperm.** Testes from individual *X. tropicalis* (A) and *X . laevis* (B) were either used fresh or frozen and thawed using the Harland method to fertilise eggs from a single female of known good quality. A-I on the X-axis refers to sperm samples, each from a different male. The experiment was repeated using three female frogs, to eliminate this as a possible source of variability, data from a single female are shown. Eggs that divided were counted as fertilised, and morphologically normal embryos were counted at NF32. C) The mean values from the experiments above are shown for *X. laevis* and *X. tropicalis* ± SEM. There is a significant effect of the species on the percentage fertilisation (**: *p* = 0.002) and on the percentage of normal embryos at NF32 (***: *p* = 0.0003), whether fresh or frozen sperm was used.

To compare recovery of frozen sperm for *X. laevis* and *X. tropicalis*, we tested them systematically. One unmascerated testis from each male frog was stored at 4C in 1 × MBS and the other cryopreserved as 4 aliquots using the Harland laboratory method and stored at −80C. After storage for 2 days the thawed and chilled sperm samples equivalent to 0.25 testis were used to fertilise a single clutch of eggs that has been layed into two separate petri dishes. Fertilisation rates and successful development to tailbud stage were recorded. For *X. tropicalis* all of the sperm samples, regardless of whether fresh or frozen, produced higher fertilisation rates (*p* = 0.002) and numbers of normal, NF32 embryos (*p* = 0.0003) than for *X. laevis* with the least successful rate being 5% (1/9; the remainder were >15%) and a mean of 38% (Fig. 1A, C). The situation in *X. laevis* however was very different with a significant proportion of the frozen sperm samples (3/10) failing to produce sufficient embryos that develop to NF32 to recover a line (Fig. 1B). Overall the poorer recovery of *X. laevis* sperm was reflected by a mean of 16% of the embryos developing normally at NF32 (Fig. 1C).

### 3.2 The Harland or Mansour methods are the most successful for recovery of viable sperm following long-term storage on liquid nitrogen

**Fig. 2:**
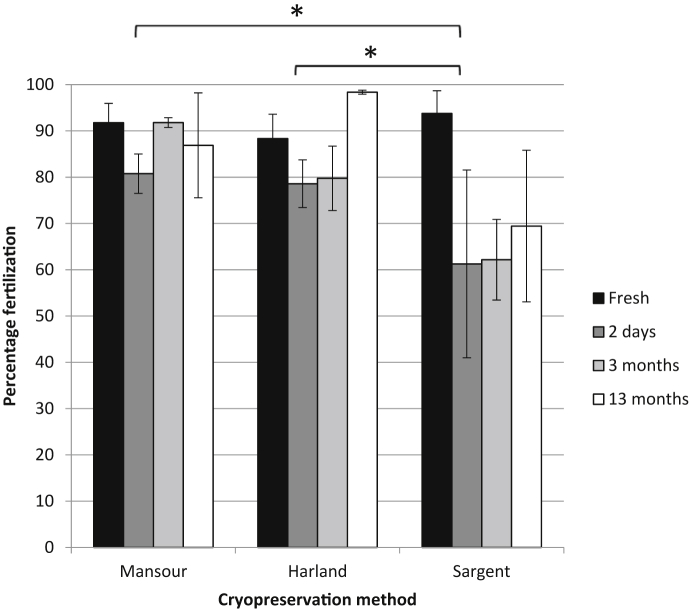
**The Mansour and Harland methods are the most effective for long-term cryopreservation of *X. laevis* sperm.** Sperm from 3 separate male frogs was either tested fresh on eggs from two females of known good quality or was tested following cryopreservation by the methods shown. Sperm were recovered after 2 days, 3 months or 13 months and fertilisation rates were recorded. Means ± SEM are shown. There is no significant difference between the Mansour and Harland methods (*p* = 0.82). There is a significant difference between the Harland and Sargent methods (*: *p* = 0.04) **and** between the Mansour and Sargent methods (*: *p* = 0.03).

Since we had found that frozen-thawed *X. laevis* sperm prepared using the Harland method were inconsistent at producing embryos, we compared the three current methods of cryopreservation. Testes from 9 males were either kept chilled or frozen on liquid nitrogen, three for each method. Their ability to fertilise two mixed clutches of eggs was tested both fresh and following 2 days of cryopreservation to ensure that the male was fertile and that the cryopreservation procedure had been successful. Cryopreserved samples were then recovered after 3 and 13 months, again their ability to fertilise clutches of eggs from 2 females was tested. There was no difference between the Harland and Mansour methods of cryopreservation (*p* = 0.82), with 80–90% mean fertilisation rates achieved and no effect of the cryopreservation time for all methods (*p* = 0.13). The Sargent method was significantly less effective than Harland (*p* = 0.04) and Mansour method (*p* = 0.03), with only 60% of embryos fertilised (Fig. 2). After 13 months the Harland method provided the highest mean fertilisation rates (Fig. 2) and we adopted this for the following experiments since it requires less specialised equipment than the Mansour method and there is no significant difference in their effectiveness.

### 3.3 *X. laevis* derived from frozen sperm develop normally

**Fig. 3:**
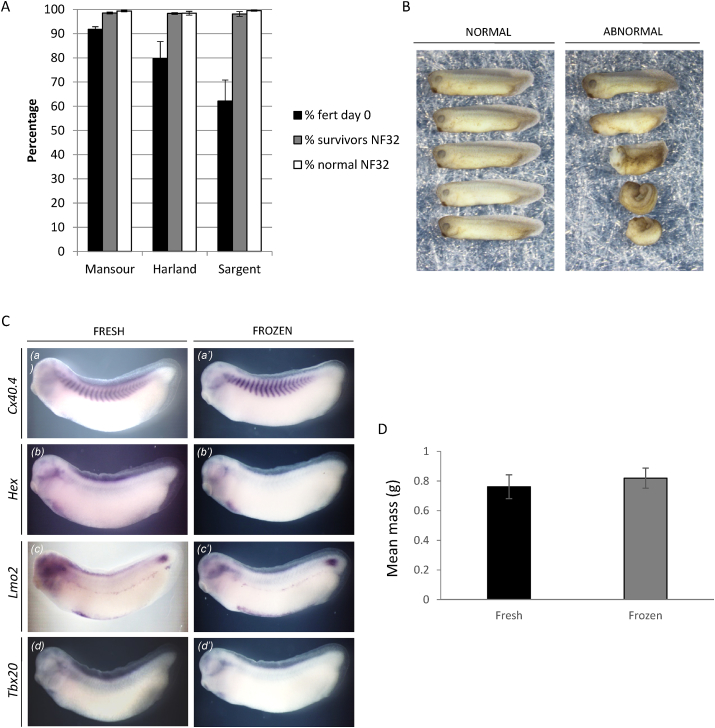
**Embryos from frozen sperm develop normally**. A) Eggs were fertilised using sperm frozen by the methods shown and stored on LN_2_ for 3 months, the number of normal and abnormal embryos was scored at NF32, normal embryo percentages are expressed as a % of the surviving embryos at NF32. Means ± SEM are shown. B) Example of normal, left, and abnormal, right, embryos fertilised with cryopreserved sperm. C) Embryos produced using fresh or frozen sperm (Harland method) were fixed at NF32 and 12 of each analysed for the expression of the markers shown by in situ hybridisation. D) Once the embryos had developed to metamorphosis they were weighed. Means ± SEM are shown.

For cryopreservation to be used routinely, it is essential that recovered sperm can produce offspring that develop normally and can in turn breed. We therefore tested whether embryos produced using thawed sperm that had been frozen for 3 months using each of the three methods had normal phenotypes at NF32. All methods produced more than 90% phenotypically normal embryos at NF32 (the percentage shown is of surviving embryos not original, fertilised eggs; Fig. 3A and B). We also tested whether a restricted set of marker genes for somites, liver precursors, blood and cardiac muscle was normally expressed in embryos derived from frozen sperm (Fig. 3C); they were expressed normally. Finally, we grew embryos up to metamorphosis (NF59) and weighed them to test whether there was a longer-term effect of cryopreservation on developmental success; there was no difference between the masses of metamorphs from fresh or frozen sperm (Fig. 3D). These metamorphs were then allowed to grow to adulthood and used alongside other *Xenopus* in the EXRC for routine embryo production in a blinded experiment; there was no difference noted between using these adults (n = 18) and any others in terms of breeding success, even when pairs with both frogs derived from frozen sperm were used.

### 3.4 Storage in liquid nitrogen or at −80C are similarly effective for *X. laevis* sperm cryopreservation

**Fig. 4:**
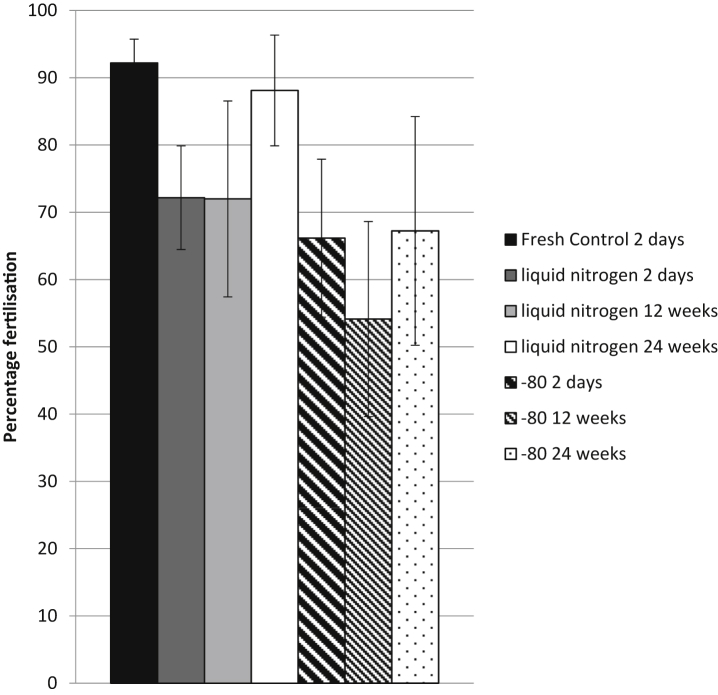
**There is no difference in sperm activity between cryopreserved sperm stored in liquid nitrogen and cryopreserved sperm stored at -80C.** Eggs were fertilised using sperm frozen by the Harland method and stored on LN_2_ or at −80C for 2 days, 12 weeks or 24 weeks. Means ± SEM are shown. The storage method does not have an effect on the fertility (*p* = 0.08), and neither does the time stored (*p* = 0.45).

We next attempted to optimise the Harland cryopreservation procedure. The first variable we tested was the effect of storage conditions on fertilisation rates using cryopreserved sperm. Aliquots of identical sperm samples prepared from three frogs using the Harland method were stored either on liquid nitrogen or at −80C. The samples were tested fresh and then after 2 days of cryopreservation to ensure that the sperm were initially active and that the cryopreservation was successful. Their activity was then compared after 12 and 24 weeks. There was no effect of the method (*p* = 0.08) and the time of cryopreservation (*p* = 0.45) on the percentage fertilisation (Fig. 4).

### 3.5 Maceration method does not affect the activity of frozen-thawed sperm

We next tested whether the methods of maceration affected the fertilisation rates that could be achieved using the cryopreserved sperm samples produced. We compared homogenising testes in a microcentrifuge tube with a plastic pestle (EXRC method) or tearing the testes apart with two pairs of forceps (NXR method) (Fig. S1). The choice of maceration technique had no effect on either fertilisation rate (*p* = 0.86) or normal development to NF32 (*p* = 0.34).

### 3.6 Dividing sperm into 8, rather than 4, aliquots for cryopreservation does not inhibit fertilisation rates or subsequent development

Previous papers have described dividing each testis into 4–5 samples (Sargent and Mohun) or 8 (Mansour et al.), so we tested directly whether that had an effect on sperm activity. For each of 3 frogs one testis was macerated and frozen in 4 × 250 μL aliquots and the other was frozen as 8 × 125 μL aliquots. After 13 months' storage in liquid nitrogen these were thawed and used to fertilise eggs from 2 different females (Fig. S2). Whether a testis was divided into 4 or 8 did not affect fertilisation success (*p* = 0.12), suggesting that we are using an excess of sperm even when dividing a testis into 8.

### 3.7 Delays to freezing and thawing do not affect fertilisation rates

We tested whether a delay, either between maceration of the testes and freezing, or between thawing sperm and adding them to the eggs, influenced fertilisation rates and normal development, as assessed at NF32 (Fig. S3). Delays of up to 20 min prior to freezing had no effect on either fertilisation (*p* = 0.18) or survival of embryos to NF32 (*p* = 0.95). Delays of up to 20 min between thawing of sperm and its addition to eggs had no effect on either fertilisation (*p* = 0.06) or survival of embryos to NF32 (*p* = 0.56). Finally, we kept macerated testes in either 1 × or 0.1 × MBS for up to 20 min prior to fertilisation to test whether prolonged activation affected sperm activity (Fig. S4). There was no significant effect on fertilisation rate (*p* = 0.88) or normal survival to NF32 (*p* = 0.18).

## 4 Discussion

Recovery of *X. laevis* sperm from cryopreservation is inconsistent. Here we have shown that this is not due to small differences in technical replication. We have tested the parameters that can be altered, either deliberately or accidentally, in the freeze-thaw method and show that varying them does not have any significant effect on either fertilisation rates or the survival of normal embryos to NF32. Having eliminated these technical reasons as underpinning the variability seen we conclude that the variation is largely due to differences between individual animals; an example can be seen in Fig. S1 where an unusually small fraction of embryos develops normally to NF32. This is well documented in other species [55], [56]. During the course of this study, individuals giving such poor sperm samples that we could not guarantee to recover GA *Xenopus laevis* lines from cryopreservation stopped occurring; we propose that this is due to the increased health of our colonies.

An unexpected result, that leaving sperm for 20 min at room temperature did not affect fertilisation rates even when the incubation was in 0.1 × MBS (i.e. activated sperm), suggests that *X. laevis* sperm retain the capability to fertilise under optimal conditions even when they have significant plasma membrane and DNA damage [44]. The lack of an effect of activation on the state of frozen-thawed sperm is in agreement with Morrow et al. [44].

The adults derived from frozen-thawed *X. laevis* sperm are indistinguishable in terms of embryo production from others in a large breeding colony and this can give the community confidence to store GA *X. laevis* lines as frozen sperm. The detailed sperm cryopreservation method is given in Supplementary Table 1 and will also be available as a live document constantly updated with any improvements on both the EXRC (https://xenopusresource.org/) and NXR (http://www.mbl.edu/xenopus/) websites. The study published here makes it clear that the method is very robust and that no step is, for example, time critical. This, together with the fact that sperm can be stored for long periods at −80C, a facility that any lab will have access to, means that this method can be widely adopted.

This has important implications for the *Xenopus* research community; for dominant transgenic lines it will be possible to send aliquots of frozen sperm rather than live males out to labs requiring that line. Since 16 aliquots of sperm can be taken from each male it will be possible for a laboratory using a line regularly not to keep the animals, but to keep several male equivalents of sperm as aliquots in the freezer. This will reduce the costs to *Xenopus* users, reduce the number of males required (often a transgenic male is killed to produce sperm for one or two experiments due to the instability of the sperm within the dissected testes, rather than 16 experiments) and decrease the stress of travel to which males may be exposed. Together these are a significant contribution to the 3Rs [57].
