# Supplementary material for: An optimized method for cryogenic storage of Xenopus sperm to maximise the effectiveness of research using genetically altered frogs
Source: Theriogenology. 2017 Apr 1;92:149–55. doi: 10.1016/j.theriogenology.2017.01.007 (PMC5340284; doi:10.1016/j.theriogenology.2017.01.007)
Supplement: Supplementary file 1 [file mmc1.pptx]

## Slide 1
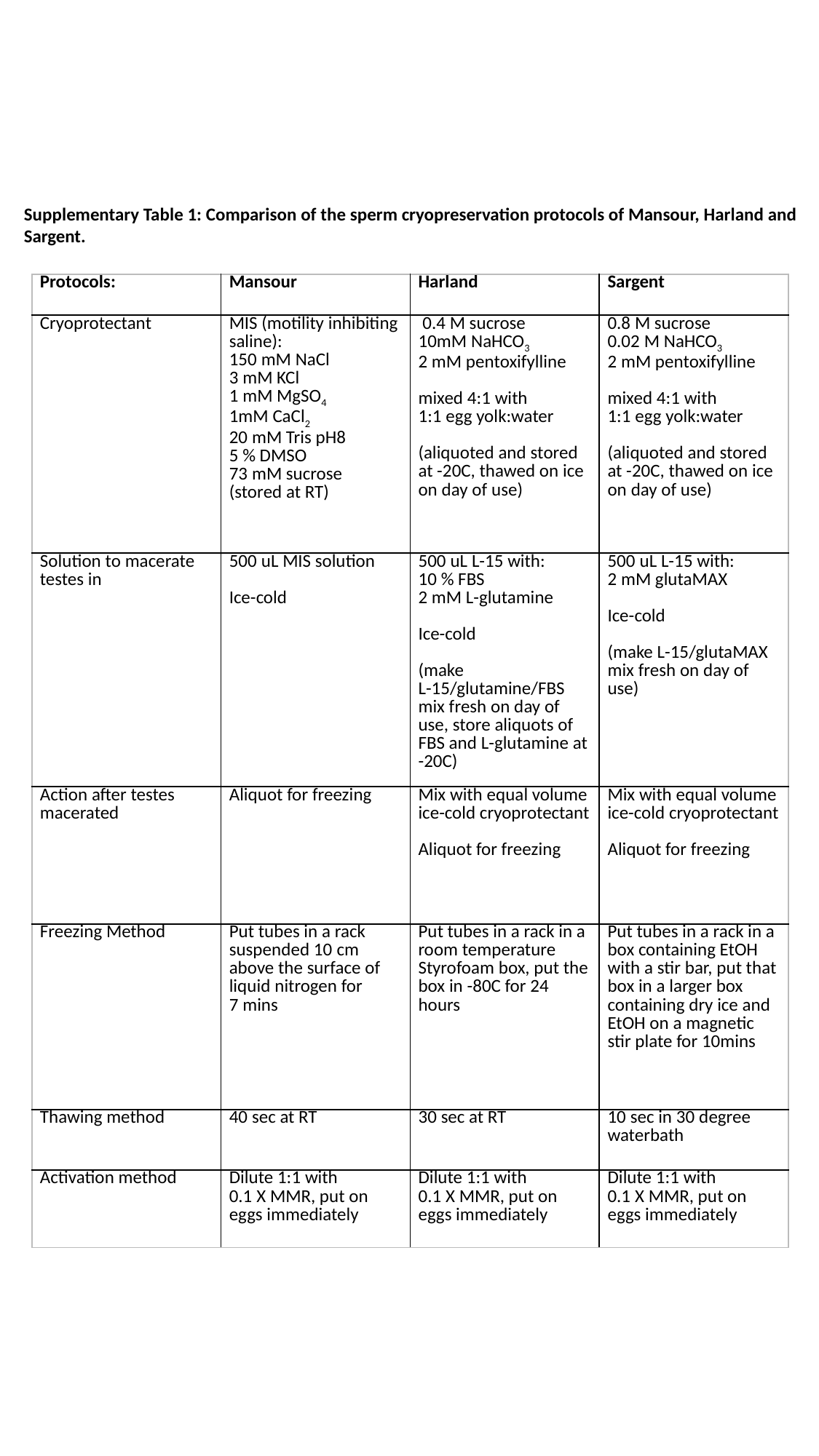

Supplementary Table 1: Comparison of the sperm cryopreservation protocols of Mansour, Harland and Sargent.
| Protocols: | Mansour | Harland | Sargent |
| --- | --- | --- | --- |
| Cryoprotectant | MIS (motility inhibiting saline): 150 mM NaCl 3 mM KCl 1 mM MgSO4 1mM CaCl2 20 mM Tris pH8 5 % DMSO 73 mM sucrose (stored at RT) | 0.4 M sucrose 10mM NaHCO3 2 mM pentoxifylline   mixed 4:1 with 1:1 egg yolk:water   (aliquoted and stored at -20C, thawed on ice on day of use) | 0.8 M sucrose 0.02 M NaHCO3 2 mM pentoxifylline   mixed 4:1 with 1:1 egg yolk:water   (aliquoted and stored at -20C, thawed on ice on day of use) |
| Solution to macerate testes in | 500 uL MIS solution Ice-cold | 500 uL L-15 with: 10 % FBS 2 mM L-glutamine Ice-cold   (make L-15/glutamine/FBS mix fresh on day of use, store aliquots of FBS and L-glutamine at -20C) | 500 uL L-15 with: 2 mM glutaMAX Ice-cold   (make L-15/glutaMAX mix fresh on day of use) |
| Action after testes macerated | Aliquot for freezing | Mix with equal volume ice-cold cryoprotectant   Aliquot for freezing | Mix with equal volume ice-cold cryoprotectant   Aliquot for freezing |
| Freezing Method | Put tubes in a rack suspended 10 cm above the surface of liquid nitrogen for 7 mins | Put tubes in a rack in a room temperature Styrofoam box, put the box in -80C for 24 hours | Put tubes in a rack in a box containing EtOH with a stir bar, put that box in a larger box containing dry ice and EtOH on a magnetic stir plate for 10mins |
| Thawing method | 40 sec at RT | 30 sec at RT | 10 sec in 30 degree waterbath |
| Activation method | Dilute 1:1 with 0.1 X MMR, put on eggs immediately | Dilute 1:1 with 0.1 X MMR, put on eggs immediately | Dilute 1:1 with 0.1 X MMR, put on eggs immediately |

## Slide 2
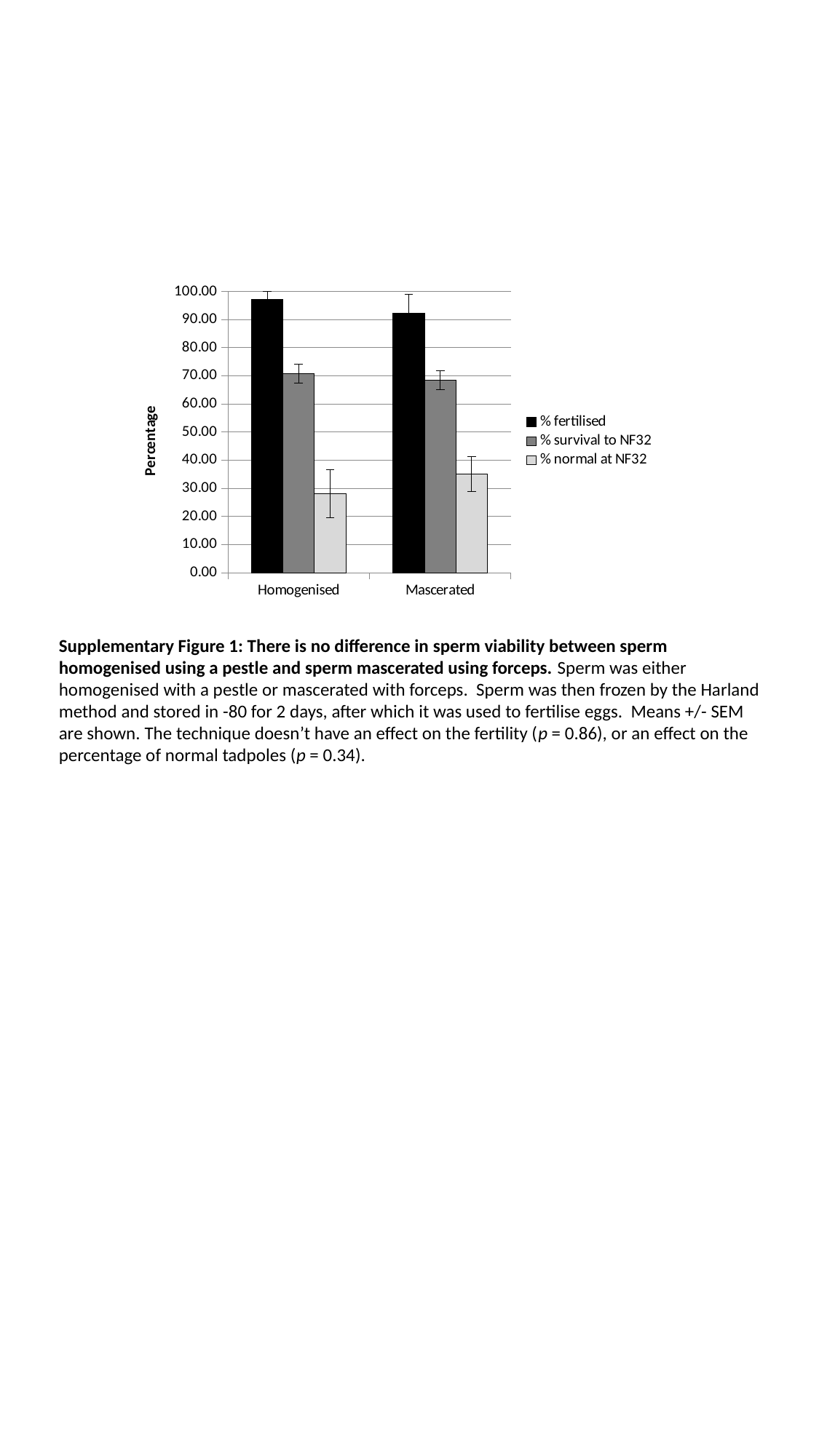

### Chart
| Category | % fertilised | % survival to NF32 | % normal at NF32 |
|---|---|---|---|
| Homogenised | 97.25085910652894 | 70.65387016229693 | 27.98245614035088 |
| Mascerated | 92.11601307189542 | 68.43835242771411 | 35.02916452221639 |Supplementary Figure 1: There is no difference in sperm viability between sperm homogenised using a pestle and sperm mascerated using forceps. Sperm was either homogenised with a pestle or mascerated with forceps. Sperm was then frozen by the Harland method and stored in -80 for 2 days, after which it was used to fertilise eggs. Means +/- SEM are shown. The technique doesn’t have an effect on the fertility (p = 0.86), or an effect on the percentage of normal tadpoles (p = 0.34).

## Slide 3
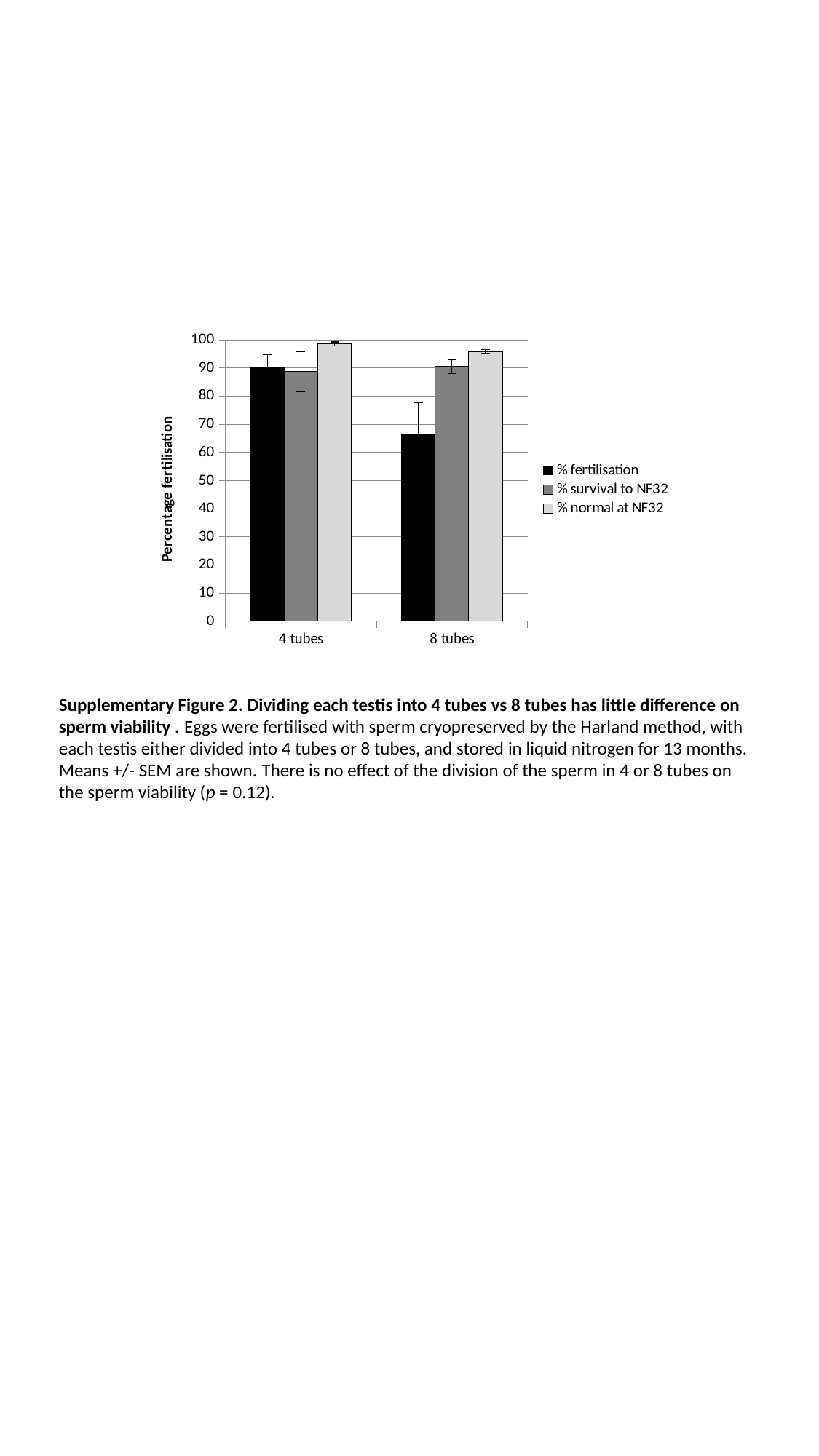

### Chart
| Category | % fertilisation | % survival to NF32 | % normal at NF32 |
|---|---|---|---|
| 4 tubes | 90.16149516890007 | 88.7385404613929 | 98.498802985495 |
| 8 tubes | 66.14567694487356 | 90.53321109848152 | 95.79782285346715 |Supplementary Figure 2. Dividing each testis into 4 tubes vs 8 tubes has little difference on sperm viability . Eggs were fertilised with sperm cryopreserved by the Harland method, with each testis either divided into 4 tubes or 8 tubes, and stored in liquid nitrogen for 13 months. Means +/- SEM are shown. There is no effect of the division of the sperm in 4 or 8 tubes on the sperm viability (p = 0.12).

## Slide 4
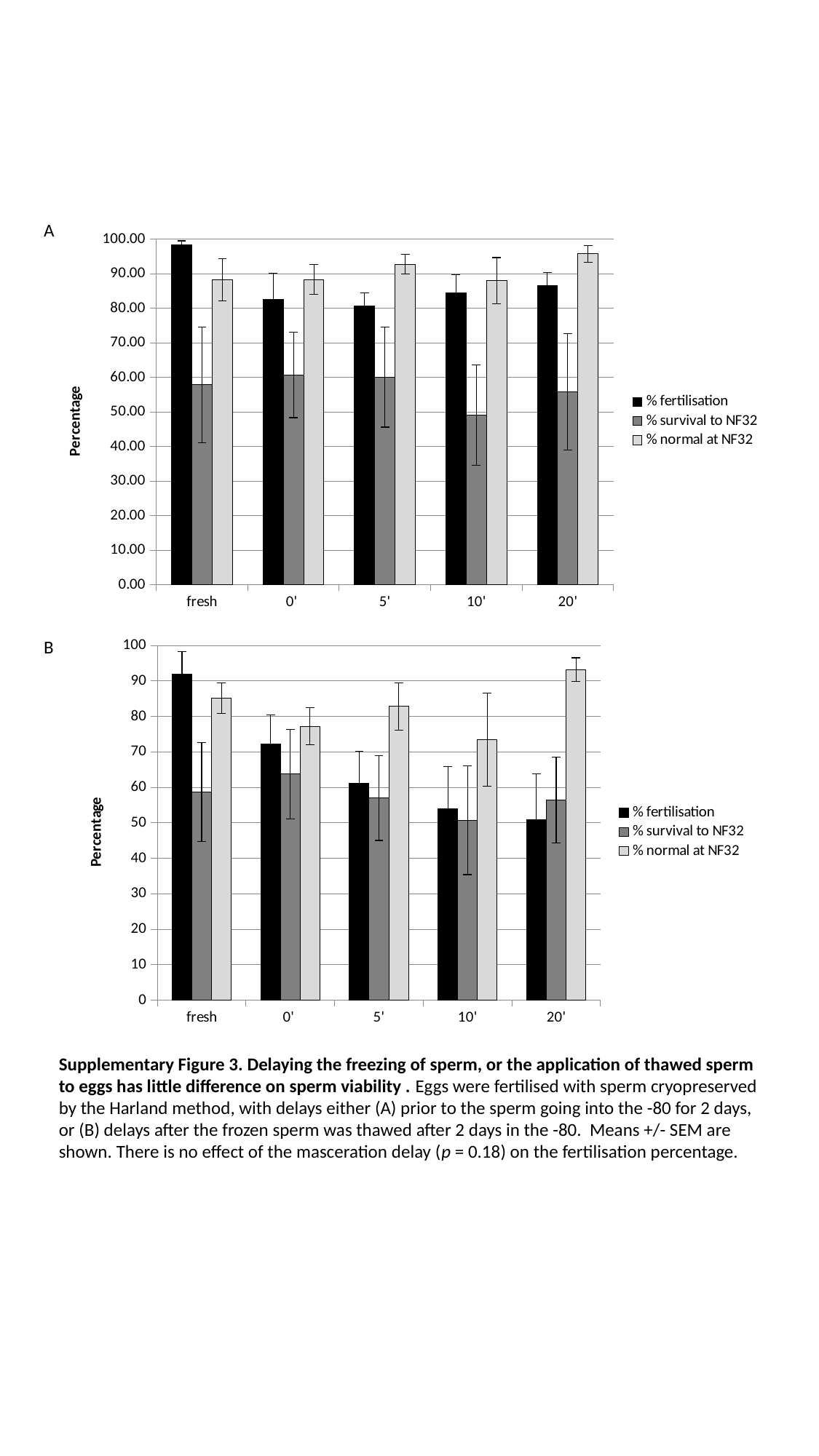

A
### Chart
| Category | % fertilisation | % survival to NF32 | % normal at NF32 |
|---|---|---|---|
| fresh | 98.4906185665199 | 57.900879004382 | 88.3611633272261 |
| 0' | 82.61518850132143 | 60.74746739952207 | 88.35346695316345 |
| 5' | 80.72637874839299 | 60.08220083217436 | 92.80529651299715 |
| 10' | 84.40300471008239 | 49.1222550805807 | 88.05065132042249 |
| 20' | 86.61847380809529 | 55.79624563812136 | 95.80689499415635 |B
### Chart
| Category | % fertilisation | % survival to NF32 | % normal at NF32 |
|---|---|---|---|
| fresh | 91.96003839339932 | 58.68464880641717 | 85.15590540523262 |
| 0' | 72.14632075419611 | 63.78155075411235 | 77.25310205947571 |
| 5' | 61.13463566888537 | 57.03659024241842 | 82.81516810664976 |
| 10' | 53.99145705813977 | 50.72842489121555 | 73.39501775080335 |
| 20' | 50.83801599373167 | 56.38931185034809 | 93.19009206597028 |Supplementary Figure 3. Delaying the freezing of sperm, or the application of thawed sperm to eggs has little difference on sperm viability . Eggs were fertilised with sperm cryopreserved by the Harland method, with delays either (A) prior to the sperm going into the -80 for 2 days, or (B) delays after the frozen sperm was thawed after 2 days in the -80. Means +/- SEM are shown. There is no effect of the masceration delay (p = 0.18) on the fertilisation percentage.

## Slide 5
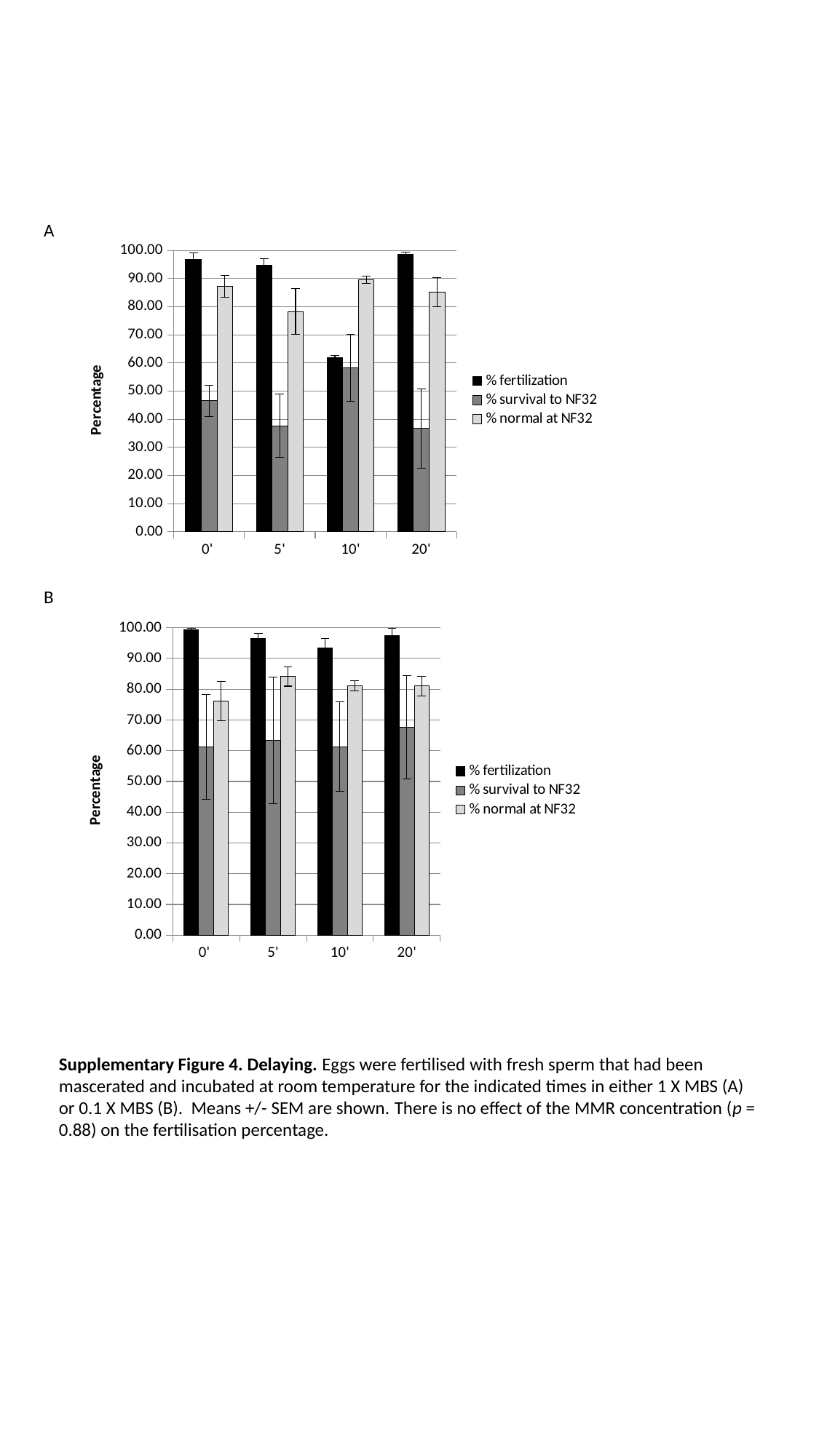

A
### Chart
| Category | % fertilization | % survival to NF32 | % normal at NF32 |
|---|---|---|---|
| 0' | 96.83068195831792 | 46.57142857142847 | 87.24867724867725 |
| 5' | 94.63877201184322 | 37.67917346984817 | 78.30513784461127 |
| 10' | 62.0 | 58.29491591658253 | 89.5642895642896 |
| 20' | 98.62363620494021 | 36.7363727363728 | 85.2325581395346 |B
### Chart
| Category | % fertilization | % survival to NF32 | % normal at NF32 |
|---|---|---|---|
| 0' | 99.36219906575639 | 61.17015098722415 | 76.1617537231472 |
| 5' | 96.49259259259243 | 63.41971584023104 | 84.10968849402875 |
| 10' | 93.31797965177196 | 61.3170687383581 | 81.11111111111111 |
| 20' | 97.3601723969044 | 67.546034507757 | 81.003801264196 |Supplementary Figure 4. Delaying. Eggs were fertilised with fresh sperm that had been mascerated and incubated at room temperature for the indicated times in either 1 X MBS (A) or 0.1 X MBS (B). Means +/- SEM are shown. There is no effect of the MMR concentration (p = 0.88) on the fertilisation percentage.
